# Paclitaxel is necessary for improved survival in epithelial ovarian cancers with homologous recombination gene mutations

**DOI:** 10.18632/oncotarget.9373

**Published:** 2016-05-14

**Authors:** Stephanie Jean, Jiaqi Li, Dionyssios Katsaros, Bradley Wubbenhorst, Kara N. Maxwell, Lauren Fishbein, Michael W. McLane, Chiara Benedetto, Emilie Marion Canuto, Nandita Mitra, Lin Zhang, Katherine L. Nathanson, Janos L. Tanyi

**Affiliations:** ^1^ Division of Gynecologic Oncology, Department of Obstetrics and Gynecology, Perelman School of Medicine, University of Pennsylvania, Philadelphia, Pennsylvania, USA; ^2^ Department of Biostatistics and Epidemiology, Perelman School of Medicine, University of Pennsylvania, Philadelphia, Pennsylvania, USA; ^3^ Department of Surgical Sciences, Gynecologic Oncology, Azienda Ospedaliero-Universitaria Città della Salute, Turin, Italy; ^4^ Division of Translational Medicine and Human Genetics, Department of Medicine, Perelman School of Medicine, University of Pennsylvania, Philadelphia, Pennsylvania, USA; ^5^ Division of Hematology and Oncology, Department of Medicine, Perelman School of Medicine, University of Pennsylvania, Philadelphia, Pennsylvania, USA; ^6^ Division of Endocrinology, Diabetes, and Metabolism, Department of Medicine, Perelman School of Medicine, University of Pennsylvania, Philadelphia, Pennsylvania, USA; ^7^ Ovarian Cancer Research Center, University of Pennsylvania, Philadelphia, Pennsylvania, USA; ^8^ Abramson Cancer Center, Perelman School of Medicine, University of Pennsylvania, Philadelphia, Pennsylvania, USA

**Keywords:** ovarian cancer, massively parallel sequencing, clinical molecular genetics, homologous recombination, paclitaxel

## Abstract

**PURPOSE:**

To investigate the impact of somatic mutations in homologous recombination (HR) genes on the chemotherapeutic response and survival of patients with epithelial ovarian cancer (EOC).

**EXPERIMENTAL DESIGN:**

We performed targeted massively parallel sequencing of tumor DNA from 158 patients with EOC. We associated adjuvant chemotherapy and clinical outcome with mutations in selected genes, focusing on those encoding HR proteins.

**RESULTS:**

HR mutations were found in 47 (30%) tumors. We did not detect an overall survival (OS) difference in advanced stage patients whose tumors had HR mutations compared to those without (median OS of 49.6 months (95% CI 29.9-57.7) *vs*. 43.3 months (95% CI 31.9-75.47), *p* = 0.87). However, when stratified by chemotherapy regimen, patients whose tumors had TP53 and HR mutations demonstrated a marked survival advantage when treated with platinum and paclitaxel *vs*. platinum +/− cyclophosphamide (median OS of 90 months (95% CI 50-NA) *vs*. 29.5 months (95% CI 17.7-50.5), *p* = 0.0005).

**CONCLUSIONS:**

Previous studies demonstrating a survival advantage for EOC patients with somatic HR mutations have been conducted with almost universal use of both platinum and paclitaxel. Our study is the first to our knowledge to compare cohorts with somatic HR gene mutations treated with and without paclitaxel containing platinum regimens. The survival benefit attributed to the platinum sensitivity of HR deficient ovarian cancers may depend upon the combined use of paclitaxel.

## INTRODUCTION

Epithelial ovarian cancer (EOC) remains the most deadly gynecologic cancer in the United States, and the fifth leading cause of cancer deaths in women [1]. Although the vast majority of EOC cases are sporadic, up to 20% are associated with inherited cancer susceptibility syndromes, most due to germline mutations in the homologous recombination (HR) genes *BRCA1* and *BRCA2* (*BRCA1/2*) [2]. EOC patients with inherited mutations in *BRCA1/2* have a longer survival than patients with sporadic disease, believed to be in part due to increased sensitivity of the *BRCA1/2* tumors to platinum-based therapy and other agents such as pegylated liposomal doxorubicin [3, 4]. Several studies suggest that sporadic ovarian carcinomas with somatic mutations in *BRCA1/2* also display differential response to platinum treatment with improved survival [5–7]. Given this differential response, there is ongoing interest in expanding on the original concept of phenotype “BRCAness” [8], and identifying sporadic ovarian cancers with defects in the HR pathway that may benefit from platinum and PARP-inhibitor therapy [9, 10].

TCGA data demonstrate that up to 50% of EOC cases have either germline or somatic defects in HR genes [6]. Platinum based chemotherapy was initiated in the mid 1980s and the high frequency of HR gene mutations may partially explain the improved treatment outcomes after initiation of this therapy [11, 12]. The combination of platinum with paclitaxel was established in the mid 1990s as the most effective and durable treatment of EOC, and has remained the standard of care [13–16]. All recent studies examining genetic/genomic correlates of survival of EOC patients are comprised of patients treated with both adjuvant platinum and paclitaxel [3, 5, 7, 9].

We examined cohorts of patients treated with platinum before and after the establishment of combination therapy with paclitaxel as first-line therapy of EOC. Our goal was to determine if survival differed based on tumor mutations and chemotherapy regimen. Currently, emphasis on treatment response for EOC is placed on platinum sensitivity, which dictates management with second and third line therapy as well as clinical trial eligibility. We aimed to examine the response of EOC patients with somatic HR gene mutations and delineate the contribution of paclitaxel added to a platinum-based regimen.

## RESULTS

### Clinical cohort characteristics

After two samples were removed due to poor sequencing quality, the total cohort included 158 patients, of which 144 (91%) had available clinical data. Of the 144 EOC patients, 91 had serous, 44 had endometrioid and 9 had clear cell histology (Table 1).

**Table 1:** Clinical and mutational characteristics of subjects by chemotherapy regimen

	All subjects with clinical data	Received platinum + paclitaxel	Received platinum +/− cyclophos-phamide	P-value^†^
Mean age (years)	57.9	55.7	59.2	0.093
Histology				
High-grade serous carcinoma Low-grade serous carcinoma High-grade endometrioid carcinoma Low-grade endometrioid carcinoma Clear cell carcinoma	76 (52.8%) 15 (10.4%) 28 (19.4%) 16 (11.1%) 9 (6.3%)	43 (64.2%) 8 (11.9%) 13 (19.4%) 3 (4.5%) 0 (0.0%)	26 (48.1%) 2 (3.7%) 12 (22.2%) 7 (13.0%) 7 (13.0%)	0.003
Stage				
I II III IV	35 (24.3%) 9 (6.3%) 88 (61.1%) 12 (8.3%)	6 (9.0%) 4 (6.0%) 51 (76.1%) 6 (9.0%)	14 (36.0%) 3 (5.6%) 32 (59.3%) 5 (9.3%)	0.092
Debulking status				
Optimal Suboptimal Unknown	80 (55.6%) 62 (43.1%) 2 (2.4%)	35 (52.2%) 31 (46.3%) 1 (1.4%)	27 (50%) 27 (50%) 0 (0.0%)	0.741
Tumor mutation status				
HR pathway mutation HR wild type *TP53* mutation *TP53* wild type HR and *TP53* mutation Wild type for both HR and *TP53*	47 (32.6%) 97 (67.4%) 81 (56.3%) 63 (43.8%) 31 (21.5%) 113 (78.5%)	28 (41.8%) 39 (58.2%) 41 (61.2%) 26 (38.8%) 18 (26.9%) 49 (73.1%)	14 (25.9%) 40 (74.1%) 31 (57.4%) 23 (42.6%) 11 (20.4%) 43 (49.6%)	0.068 0.673 0.405
**Total**	**144**	**67**	**54**	

HR = Homologous recombination

† P-value of the comparison between subjects who received platinum + paclitaxel vs. those who received platinum +/− cyclophosphamide. P-values in Table 1 are calculated using the two sample student T test and Chi-square test. Fisher's exact tests were used for categorical variables with small counts.

Forty-four of the 144 patients with clinical data had stage 1 or 2 disease, and the remaining 100 patients had stage 3-4 disease (70%). All patients who received adjuvant chemotherapy (*n* = 121) received a platinum based regimen. Of the advanced stage patients, thirty-seven (37%) received first-line adjuvant treatment with platinum alone or with platinum plus cyclophosphamide. Fifty-seven patients (57%) received primary adjuvant treatment with platinum plus paclitaxel. Five patients (5%) received platinum, epirubicin, and cyclophosphamide for primary adjuvant therapy. An additional four patients (4%) received a combination of platinum, paclitaxel, and epirubicin for their primary adjuvant treatment. One patient with a synchronous endometrial cancer received primary adjuvant treatment with platinum plus doxorubicin.

### Somatic mutations and variants

From 158 tumors, we identified 224 deleterious variants in 34 genes, including 116 truncating mutations (50 nonsense, 66 frameshift) and 99 missense mutations. *TP53* (*n* = 90) and *BRCA1* (*n* = 26) mutations were the most frequent, followed by *PTEN* (*n* = 18), *MSH6* (*n* = 14), *NF1* (*n* = 12), *RB1* (*n* = 9), *BRCA2* (*n* = 8), and *ATM* (*n* = 5) (Supplemental Table 1). Loss-of-function mutations in *TP53* were found in 89 samples (56%). *TP53* mutations were highly correlated with high-grade histology (Odds Ratio [OR] 6.5 with 95% CI: 2.57-16.4, p-value < 0.0001), including 53 (65.4%) high-grade serous, and 21 (24.7%) high-grade endometrioid tumors. Tumors with *TP53* mutations also included four (4.9%) low-grade serous and three (3.7%) low-grade endometrioid tumors. The majority of *TP53* mutations were missense mutations within exons 5-8, and previously described as pathogenic. We also identified 11 previously unreported frameshift mutations in *TP53*.

Deleterious variants in genes encoding proteins of the HR pathway were detected in the ovarian cancers of 47 patients (28%). Thirty mutations (63.8%) were found in high-grade serous; three (6.4%) in low-grade serous; 10 (21.3%) in high-grade endometrioid; and four (8.5%) in low-grade endometrioid tumors. Mutations in the HR pathway were identified in 15 different genes: 26 mutations in *BRCA1* (58% of the HR gene variants), 8 in *BRCA2* (18%), 5 in *ATM* (11%), 3 in *RBBP8* (7%), 3 in *PALB2* (7%), 2 in *BRCC3* (4%), and 1 each in *BAP1, BARD1, BLM, BRIP1, CHEK2, RAD50, RAD51C, RAD51D,* and *XRCC2* (2% each). Thirty-one of the 47 patients with HR pathway mutations (66%) had co-occurring *TP53* mutations.

We detected 26 loss-of-function *BRCA1* mutations (6 nonsense, 12 frameshift, 7 missense, and 1 splicing). In addition, one tumor had two distinct frameshift insertions *in cis*, (c.5077_5078insTCATT, c.5080_5081insC), located three base pairs apart. The combined insertions added six base pairs, returning the sequence in frame. The presence of a secondary mutation restoring a functional reading frame is a reported mechanism of resistance to platinum therapy [17] and observed in recurrent carcinomas after adjuvant chemotherapy with platinum-based regimens [18, 19]. Interestingly, this patient had no known prior history of receiving chemotherapy or radiation.

In 12 patients' tumors, we detected 16 mutations in mismatch repair genes *MSH2* and *MSH6*. Over half of these patients' tumors (*n* = 7) had serous histology; three unique *MSH6* variants were detected in a single tumor (p.F573fs, p.C694X p.T1085fs). In 12 patients' tumors, we detected 18 mutations in *PTEN*. The majority of these tumors (*n* = 8) had serous histology. Two of the *PTEN* mutations were novel frameshift insertions, while the remaining mutations have been described previously.

### Copy number analysis

**Figure 1:**
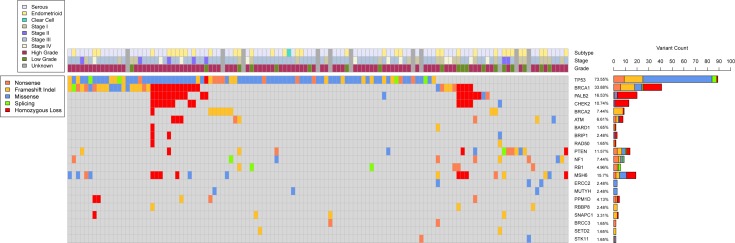
Somatic genomic landscape of epithelial ovarian cancer This figure includes all genes with deleterious mutations that were detected in at least two distinct tumors. Mutation count, type, and associated clinical data are included, as well as instances of detected homozygous copy loss.

The profile of copy number variation (CNV) resembled those previously described for high-grade serous ovarian cancers [20, 21]. Given our targeted design, we focused on gene-specific CNVs of tumor suppressor genes *TP53*, *PTEN*, *RB1*, and *NF1*, as well as genes involved in HR. We detected homozygous copy loss in one or more genes involved in HR in 23 (15%) tumors. Homozygous loss of *BRCA1*, *CHEK2*, and *PALB2* often were found simultaneously, and homozygous loss of at least two of the three co-occurred in 17 tumors (Figure 1). We detected homozygous loss of *PTEN* in three tumors, *MSH6* in nine tumors, *PPM1D* in 2 tumors, and *TP53 and SNAPC1* in one tumor each. We did not detect any instances of homozygous loss in *RB1* or *NF1*.

### Survival and outcomes analysis

Increased overall survival (OS) was significantly related to younger age at time of surgery (*p* < 0.001) and earlier stage (*p* < 0.001). We analyzed whether patients with multiple somatic mutations (19 patients with at least three detected variants) demonstrated a survival difference, and although there was a trend towards worsened survival, it was not statistically significant (*p* = 0.298).

**Figure 2:**
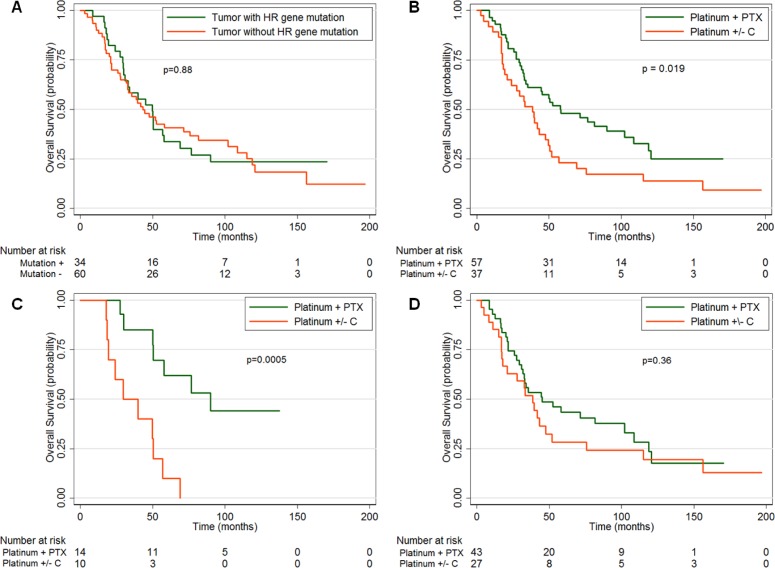
Overall survival (OS) of advanced stage patients based on mutations and/or chemotherapy regimen (Platinum + paclitaxel [PTX] or platinum +/− cyclophosphamide [C]) **A.** OS by the presence or absence of a homologous recombination (HR) gene mutation in the patient's tumor. **B.** OS by chemotherapy regimen. **C.** OS by chemotherapy regimen in patients whose tumors exhibit the “BRCAness” phenotype (both a HR and *TP53* mutation) or **D.** those without the “BRCAness” phenotype.

We performed survival analyses of our advanced stage patients who received adjuvant chemotherapy (*n* = 94). We did not find any difference in platinum sensitivity or overall survival in patients whose tumors had HR pathway mutations compared to those without such mutations (*p* = 0.84) (Figure 2A). Our patient population included 37 patients (39%) treated prior to the adoption of the combination of platinum and paclitaxel as the gold standard of first line adjuvant therapy; therefore, we investigated whether there was a difference between patients treated with platinum +/− cyclophosphamide, and platinum + paclitaxel. Our data confirm previous results that the patients who received platinum + paclitaxel had improved OS over patients who received platinum +/− cyclophosphamide [13, 14, 22] (median 57.7 months, 95% CI 33.8-102.2 *vs*. median 38.5 months, 95% CI 21-47.5, *p* = 0.019) (Figure 2B).

To investigate the survival of patients whose tumors display a phenotype of “BRCAness,” we examined the subset of patients who had high-grade tumors with HR defects by analyzing patients with stage 3 and 4 disease with tumors containing both HR and *TP53* mutations (which we term as having the “BRCAness” phenotype). Patients with tumors that contained HR and *TP53* mutations who received platinum + paclitaxel demonstrated a markedly increased OS (median 90 months, 95% CI 50-NA) compared to patients who received platinum +/− cyclophosphamide (median 29.5 months, 95% CI 17.7-50.5, *p* = 0.0005) (Figure 2C). In contrast, patients without the “BRCAness” phenotype who received platinum + paclitaxel had an OS (median 45.1 months, 95% CI 30.9-102.2) that was not significantly different than those who received platinum +/− cyclophosphamide (median 38.5 months, 95% CI 17.1-52, p = 0.36) (Figure 2D). In addition, we found that patients with the “BRCAness” phenotype trended towards a decreased median OS than patients without “BRCAness” when treated with platinum +/− cyclophosphamide (median OS of 29.5 months *vs*. 38.5 months, respectively), though this was not statistically significant (*p* = 0.442). Our sample population was too small to perform multivariate analysis; however, the two subsets did not have any statistically significant differences in age, stage, or histology.

## DISCUSSION

EOC arising in germline *BRCA1/2* mutation carriers is associated with increased chemosensitivity and a survival advantage [3, 23, 24]. Initial studies also suggest that somatic mutations in *BRCA1/2* and other DNA repair genes confer similar benefits [6, 7, 9]. Sequencing 52 genes in 158 EOC tumors, we detected mutations or deletions in 15 HR genes in the tumors of 45 patients (28%). More than half were found in *BRCA1*, followed by *BRCA2* and *ATM*. These data are similar to Pennington, et al. who examined a series of 367 patients with EOC, fallopian tube cancer, or primary peritoneal cancer and found somatic HR mutations in seven genes in 8.7% of patients and germline mutations in 11 HR genes in 24% of patients [7]. In their study, HR gene mutations (both somatic and germline) were associated with improved platinum response and OS. We did not detect a survival advantage for patients with HR pathway mutations, even though all those treated with adjuvant chemotherapy received platinum containing agents. However, a critical difference between prior studies and the current study is that our cohort included some patients treated with platinum based regimens but not paclitaxel. Thus, we evaluated whether patients whose tumors had HR deficits might respond differentially to regimens that do or do not contain paclitaxel.

In patients with high-grade tumors with HR and *TP53* mutations (“BRCAness”), we found a striking disparity in survival depending on type of chemotherapy received. When treated with platinum +/− cyclophosphamide, patients whose tumors had the “BRCAness” phenotype had no difference in OS compared to patients whose tumors did not have the “BRCAness” phenotype. Conversely, when treated with platinum + paclitaxel, patients whose tumors had the “BRCAness” phenotype had significantly improved OS as compared to patients whose tumors did not. The marked median OS improvement of 60.5 months that we observed far exceeds the 10-14 month expected based on treatment regimen alone [13, 14]. These data suggest that somatic HR mutations may be associated with an improved response to paclitaxel. Recent studies demonstrating increased platinum sensitivity in HR pathway deficient sporadic EOC cancers have been done in tumors from patients treated with both platinum and taxane combination regimens as first line adjuvant therapy [6, 7, 9]. By utilizing a cohort of patients that includes those treated prior to use of taxanes, we found that the inclusion of paclitaxel likely plays an important role in survival in patients with HR-deficient tumors.

The biological basis of platinum and PARP inhibitor sensitivity in HR-deficient ovarian tumors has been well explored in germline *BRCA1/2* mutation carriers [25], and is thought to be due to accumulation of irreparable DNA damage and loss of functional double strand break repair mechanisms, respectively, leading to cell and tumor death. However, there are only a few studies examining the effect of taxanes on ovarian cancers with HR deficits. One study examined the development of chemoresistance in ovarian cancer cell lines and found that cell lines with functional BRCA1 developed paclitaxel resistance more quickly than cell lines with non-functional BRCA1 (due to gene silencing by hypermethylation) [26]. Another study examined the ovarian cancer cell line SNU251, and found that a truncated and dysfunctional BRCA1 protein impaired sub-nuclear protein assembly required for DNA damage repair, and subsequently led to increased sensitivity of the cells to taxol therapy [27]. Although there are a limited number of clinical studies examining taxane sensitivity in germline *BRCA1/2* mutation carriers with EOC, several have demonstrated a synergy between platinum and taxanes. In a small study of recurrent platinum sensitive ovarian cancer patients, *BRCA1/2* mutation carriers demonstrated a significant response to repeat carboplatin with dose dense paclitaxel regimens, compared to sporadic patients, suggesting that this drug combination is particularly effective in *BRCA1/2*-related disease [28], and potentially by extension to HR deficient tumors. In addition, a study examining the role of taxane monotherapy in germline *BRCA1/2* mutation carriers with relapsed ovarian cancer found a significant survival benefit in patients with initially platinum-sensitive disease compared to those with platinum-resistant disease [29]. Both the pre-clinical and clinical data support our finding that EOC tumors with *BRCA1* (and other HR gene) mutations have an increased sensitivity to paclitaxel as compared to tumors without such mutations.

Recently, it has been hypothesized that the predominant cytotoxic mechanism of paclitaxel is due to chromosomal missegregation rather than strict mitotic arrest as traditionally believed [30]. Chromosomal missegregation causes genomic instability that leads to accelerated cell death. We postulate that tumors with deficient double strand break repair due to HR mutations are particularly susceptible to chromosomal missegregation, increasing their sensitivity to paclitaxel and contributing to the marked improvement in OS observed in our population.

Our study has several limitations. We have a relatively small sample size for subgroup analysis. Our capture panel is targeted rather than whole-exome or whole-genome, which would limit potential novel gene findings. Additionally, our mutational profiling is based on somatic findings without germline comparison. Some of the mutations we detected may be unrecognized germline mutations; for example, two of our patients demonstrate a *BRCA1* mutation recently described as a possible Northern Italian founder mutation [31], and one of the *MSH6* mutations we detected in five different patients has been described in a family with Muir-Torre syndrome, a variant of Lynch syndrome [32] as well as a family with early-onset colorectal cancer [33]. Nevertheless, given the practice of tumor tissue sequencing without accompanying germline testing [34], we believe our findings are applicable in the clinical setting. We recognize that the rate of *TP53* mutation in our high-grade serous tumor cohort does not reach the >95% seen in recent literature [6], even with rigorous pathology and sequencing quality control. In addition, there are histology differences in our two chemotherapy cohorts, amplified by small numbers of rare histology types. We believe that these limitations are remedied by the fact that our major analysis focuses strictly on tumors that exhibit both *TP53* and HR gene mutations, capturing the “BRCAness” phenotype we aimed to study.

Our study is the first to our knowledge to demonstrate that EOC cancer patients with somatic HR gene mutations demonstrate no improvement in, and potentially worsened, survival than those without such mutations, unless they receive taxane therapy in addition to platinum adjuvant chemotherapy. Our long term follow-up of up to 18 years (median 57.7 months), as well as our unique subset of patients treated without a taxane chemotherapeutic agent allows us to highlight the importance of paclitaxel added to a platinum-based therapy for survival in patients with somatic HR deficits. Further study of the mechanism of taxane chemo-responsiveness in somatic HR-pathway mutated EOC is warranted, and may uncover new targets for therapy and a better understanding of tumor progression and resistance in sporadic EOC. Although primary adjuvant treatment with platinum and taxanes is standard, subsequent lines of therapy after recurrence are not standardized and optimal regimens remain uncertain. Knowledge of the “BRCAness” phenotype of a sporadic EOC patient's tumor may aid in selection of second, third, and further lines of treatment, and given our data, prioritizing use of taxanes in these situations should be considered.

## MATERIALS AND METHODS

Tumor samples from patients diagnosed with primary epithelial ovarian carcinoma from December 1991 - December 2005 at the University of Turin, in Turin, Italy were included in this analysis, which was performed with IRB approval. Patients with any grade or stage of pathology-confirmed serous, endometrioid, or clear cell ovarian carcinoma were eligible. All patients underwent primary cytoreductive surgery, from which research tumor specimens were obtained with informed consent. A segment of the tumor specimen was snap frozen for subsequent DNA extraction and subsequently stored at −80°C. Patients were followed from time of initial surgery to time of death or last follow-up, and clinical information on chemotherapy regimen, treatment response, recurrence, and survival were recorded. Patients treated with adjuvant chemotherapy received platinum with or without cyclophosphamide, paclitaxel, and/or an anthracycline.

Somatic mutation screening was performed using a custom designed targeted massively parallel sequencing protocol. Genomic DNA library construction was performed using TruSeq (Illumina, San Diego, CA). Genomic DNA was sheared with fragmentase (New England Biolabs, Ipswich, MA) to achieve fragments of 180-280 bp. DNA was end repaired and ligated with adaptor-embedded indexes. Then the DNA was purified and size-selected using 2% SizeSelect E-gels (Invitrogen, Eugene, OR) and PCR enriched. DNA quantity and quality were analyzed using the Agilent 2100 Bioanalyzer (Agilent, Santa Clara, CA). High quality samples were pooled and hybridized to a custom capture library using Agilent SureSelect kits. DNA quality, size, and concentration were assessed using the Qubit^®^ 2.0 fluorometer (Life Technologies, Carlsbad, CA) and the Bioanalyzer. Library captures were sequenced using the HiSeq 2000 (Illumina) at the University of Pennsylvania Next Generation Sequencing Core.

Our custom capture panel included 52 genes, primarily tumor suppressor genes, involved in pathways implicated in ovarian and/or breast cancer susceptibility and tumorigenesis, such as HR, mismatch repair, or checkpoint inhibition. The HR pathway associated genes we assessed included *ATM, BRCA1, BRCA2, BAP1, BARD1, BLM, BRCC3, BRIP1, CHEK2, PALB2, RAD50, RAD51C, RAD51D, RBBP8, XRCC2, and XRCC3*. In addition, the following genes were also included on our panel: *ATR, BABAM1, BRE, CDH1, CDK4, CDKN2A, ERCC2, FAM175A, FANCE, JARID2, KDM6A, KEAP1, MLH1, MRE11A, MSH2, MSH6, MUTYH, NBN, NF1, PALB2, PHF3, PMS1, PMS2, PPM1D, PTEN, RAD51A, RAD51B, RB1, SETD2, SIRT1, SNAPC1, STK11, TET2, TP53, TP53BP1, UIMC1,* and *WRN.*

Alignment to human reference genome NCBI Build 37 was performed with the Burrows-Wheeler Aligner [35] and reads were analyzed using the Genome Analysis Toolkit for variant calling [36]. ANNOVAR was used for variant annotation [37, 38]. Samples were removed due to poor sequencing quality if more than 10% of targets had 0% coverage, or if at least 10x coverage was achieved in less than 50% of targets. Variant identification and classification was performed using a strict filtering and analysis pipeline that has previously been described and validated [39, 40]. Locus-specific databases, ClinVar, dbSNP, and COSMIC were utilized to help identify suspected deleterious variants, and any missense variant calls that could not be confirmed in the literature were considered variants of undetermined significance (VUS) and excluded from analysis. Pindel [41] was utilized to detect large rearrangements or indels.

Copy number analysis (CNA) was performed using ngCGH (next generation CGH) using germline DNA from a cohort of breast cancer patients as normal DNA for comparison which was matched to each EOC tumor specimen by mean target depth of coverage. CNA was visualized using Nexus Copy Number software version 7.5 Discovery Edition (BioDiscovery Inc., Hawthorne, CA). Given the targeted gene design, CNA was performed on a gene-by-gene basis, and selected tumor suppressor genes were analyzed for homozygous copy loss.

### Statistical analysis

Overall survival was determined using Kaplan Meier analysis and Log-rank tests for significance. Cox proportional hazards models were used to adjust for age, stage, and histology. We focused on advanced stage (stages III-IV) patients given the marked survival difference in this population, and assessed survival differences due to mutation status and chemotherapy regimen using Chi square and Fisher's Exact tests. All statistical analyses were performed using Stata Version 12 (College Station, TX) and R version 2.15.1 (Vienna, Austria). Statistical significance was set at alpha = 0.05 and all tests were 2-tailed.

## SUPPLEMENTARY MATERIAL TABLE




