# Supplementary material for: Paclitaxel is necessary for improved survival in epithelial ovarian cancers with homologous recombination gene mutations
Source: Oncotarget. 2016 May 14;7(30):48577–85. doi: 10.18632/oncotarget.9373 (PMC5217039; doi:10.18632/oncotarget.9373)
Supplement: Supplementary file 1 [file oncotarget-07-48577-s001.pdf]

Paclitaxel is necessary for improved survival in epithelial ovarian cancers with homologous recombination gene mutations

Supplementary Material

Supplementary Table 1: All detected deleterious mutations

For Table S1, please see the attached Excel file
